# 

**Published:** 1899-01

**Authors:** 


JANUARY, 1899.

### THE AMERICAN JOURNAL OF DENTAL SCIENCE.

LIBRARY
SURGEON GENERAL'S OFFICE
JAN.-30-1899

EDITED BY

F. J. S. GORGAS, M. D., D. D. S.
RICHARD GRADY, M. D., D. D. S.

Vol. 32. THIRD SERIES No. 9.

PUBLISHED MONTHLY.

$2.50 PER ANNUM.

BALTIMORE:
SNOWDEN & COWMAN MFG. CO., 9 W. FAYETTE ST.

LONDON:

TRUBNER & CO., 60 PATERNOSTER ROW.

Entered at the Post Office at Baltimore, Md., as Second-class Matter.

PAYABLE IN ADVANCE.

